# The European Map of Axial Spondyloarthritis: Capturing the Patient Perspective—an Analysis of 2846 Patients Across 13 Countries

**DOI:** 10.1007/s11926-019-0819-8

**Published:** 2019-03-12

**Authors:** Marco Garrido-Cumbrera, Denis Poddubnyy, Laure Gossec, David Gálvez-Ruiz, Christine Bundy, Raj Mahapatra, Souzi Makri, Laura Christen, Carlos J. Delgado-Domínguez, Sergio Sanz-Gómez, Pedro Plazuelo-Ramos, Victoria Navarro-Compán, on behalf of the EMAS Working Group

**Affiliations:** 1 Universidad de Sevilla, Seville, Spain; 2 Health & Territory Research (HTR), Centro de Investigación, Tecnología e Innovación Manuel Losada Villasante (CITIUS), Universidad de Sevilla, C/ Dr Rafael Martínez Domínguez s/n, 41013 Seville, Spain; 3 Spanish Federation of Spondyloarthritis Patient Associations (CEADE), Madrid, Spain; 4 Charité-Universitätsmedizin Berlin, Berlin, Germany; 5 German Rheumatism Research Centre, Berlin, Germany; 6 Sorbonne Université, Institut Pierre Louis d’Epidémiologie et de Santé Publique (iPLESP), Paris, France; 7 Rheumatology Department, Pitié Salpêtrière hospital, AP-HP, Paris, France; 8 Cardiff University, Cardiff, UK; 9 Ankylosing Spondylitis International Federation (ASIF), London, UK; 10 Cyprus League Against Rheumatism, Nicosia, Cyprus; 11 Novartis Pharma AG, Patient Advocacy, Basel, Switzerland; 12 IdiPaz, Hospital Universitario La Paz, Madrid, Spain

**Keywords:** Axial spondyloarthritis, Ankylosing spondylitis, Non-radiographic axial spondyloarthritis, Patient’s perspective, Burden of the disease, Shared decision-making, Europe

## Abstract

**Purpose of Review:**

Scientific research in axial spondyloarthritis (axSpA) has grown significantly. Nevertheless, the patient perspective remains insufficiently explored. Using a cross-sectional survey, the European Map of Axial Spondyloarthritis (EMAS) describes how patients living with self-reported axSpA experience their disease physically, psychologically, and socially.

**Recent Findings:**

2846 patients participated: mean age 43.9 ± 12.3 years, 61.3% female, mean disease duration was 17.2 ± 12.4 years, and 71.3% were HLA-B27 positive. Mean diagnostic delay was 7.4 ± 8.4 years. Mean BASDAI score was 5.5 ± 2.0 and 75.7% reported moderate/severe spinal stiffness throughout the day. Daily life was substantially impaired: 74.1% reported difficulties finding a job due to the disease, and 61.5% reported psychological distress.

**Summary:**

EMAS results showed long diagnostic delay and substantial physical and psychological burden, indicating important unmet needs for patients. Furthermore, axSpA restricted patients’ ability to participate in their daily routine and lead a productive work life. Understanding the patient’s perspective can improve both health outcomes and shared decision-making between patient and rheumatologist.

## Introduction

Axial spondyloarthritis (axSpA) is a chronic inflammatory disease that encompasses radiographic (traditionally known as ankylosing spondylitis [AS]) and non-radiographic (nr-axSpA) forms. This inflammatory disease can lead to chronic pain, structural damage, and disability [1]. In particular, the physical restrictions and worsening quality of life caused by the disease are closely related to the limitations that patients face in their professional, social, and family spheres [2, 3], as well as the overall impact on psychological health, not only for patients living with axSpA but also for their families [4].

The insidious nature of the disease can be misleading, as periods of apparent disease inactivity can in fact be periods of great pain, stiffness, and fatigue for patients [5]. Such discrepancy, in many cases, leads patients to feel misunderstood or disregarded [6], and therefore less likely to share their experiences with others, including their physician. Consequently, patient disengagement results in patients being less involved in medical decisions as well as poor treatment adherence, poor health outcomes [3], worse course of the disease, and quality of life [7]. For these reasons, and as indicated in the update of the ASAS/EULAR recommendations for managing axSpA, considering the patient perspective in the management of their disease and ensuring patients are sufficiently prepared to participate in discussions are critical to treatment success and good adherence [8••].

Nevertheless, recent research in the field of axSpA has largely focused on, and led to, an improved understanding of its clinical presentation and evolution of symptoms, specifically in disease activity and structural damage. Studies such as GErman SPondyloarthritis Inception Cohort (GESPIC) [9], the Outcome in AS International study (OASIS) conducted in France, Belgium and the Netherlands [10], the DEvenir des Spondylarthropathies Indifférenciées Récentes (DESIR) cohort in France [11], and the ESPeranza program for diagnosing early spondyloarthritis in Spain [12] have helped to strengthen scientific evidence and transform clinical practice. Although clinical studies often collect data on functional limitation, psychological distress, or working impact, they invariably do so using tools created with a clinical and not wholly patient perspective, thereby missing essential aspects relevant to patients and important to their optimal management.

European Map of Axial Spondyloarthritis (EMAS) aimed to generate evidence on patient-reported aspects of axSpA using a questionnaire developed in collaboration with patients, the Ankylosing Spondylitis International Federation (ASIF), clinical academic experts, describing how patients self-reporting as axSpA experience their disease from a physical, psychological, and social perspective and how they are managed within healthcare systems. We anticipate that the data gathered will help to highlight current unmet needs, including the need for early diagnosis, as well as inform personalized long-term disease management plans and treatment goals and to ultimately improve quality of life and optimize clinical outcomes for patients.

## Methods

### Design and Survey Development

EMAS was a cross-sectional survey of patients self-reporting as axSpA from Austria, Belgium, France, Germany, Italy, the Netherlands, Norway, Russia, Slovenia, Sweden, Switzerland, the UK, and Spain. The survey was adapted from the Spanish Atlas of Axial Spondyloarthritis 2017 [13], a pilot survey held from January to March 2016 led by the Health & Territory Research group of the University of Seville and including representatives from the Spanish Society of Rheumatology, the Spanish Federation of Spondyloarthritis Patient Associations (CEADE), the Max Weber Institute, and Novartis Farmacéutica Spain.

The EMAS questionnaire was originally developed in Spanish and subsequently translated into English followed by Dutch, French, German, Italian, Russian, Swedish, and Slovenian. Prior to the start of data collection, participating countries were asked to assess and modify questions for local relevance, with guidance to only make essential changes in order to maintain consistency on a pan-European level. Eight questions were removed from the original Atlas of Axial Spondyloarthritis in Spain 2017 survey template, as they were country-specific. The final patient questionnaire included 108 items related to 12 different areas: socio-demographic and anthropometric characteristics, disability assessment, work life, daily life, lifestyle habits, diagnostic journey, healthcare resource use, treatment, comorbidities (including extra-articular manifestations), psychological health, disease outcomes, and patient disease-related attitudes and treatment goals (see Table 1).

**Table 1:** Areas, variables, and measurements/categories included within the EMAS patient questionnaire

Area	Variable	Measurement/categories
Socio-demographic and anthropometric characteristics	Country of residency	Name of the country
	Age	Years
	Gender	Female, male
	Marital status	Single, married, separate/divorced, widowed
	Number of children, number of family members	Numerical
	Relationship status	Yes, no
	Educational level	No schooling, primary, high school, university
	Household income level	Euros per month; household income level per capita, calculated dividing this value by number of household members
	Membership to patients association	Yes, no
	Weight and height	Kg and cm
		Body mass index, calculated from these two indicators
Disability assessment	Assessment of disability	Yes, no
	Degree of disability	Yes, no
	Social security benefits	Yes (type of security benefit), no
Work life	Employment status	List of 8 professional status
	Main occupation	List of 11 occupations
	Hours per week in main occupation	Numerical
	Work-related issues	List of 7 work-related issues: asked for days off (number of days), took sick leave (number of days), reduced working hours (number of hours), missed work for doctor appointments, difficulty fulfilling working hours, changed work shift, suffering of professional life (yes, no)
	Employment status due to axSpA	Yes, no
	Job loss due to axSpA	Yes, no
Daily life	Functional limitation in daily activities	Degree of functional limitation in 18 daily life activities
	Help needed in daily activities	Frequency of help needed in 18 daily life activities
	Impact on social relationships	List of 5 social relationship (much better than before, better than before, same as before, worse than before, much worse than before)
	Frequency of leisure/cultural activites	List of 5 leisure/cultural activities (much more than before, more than before, same as before, less than before, much less than before)
	Adaptations since disese onset	List of 5 adaptations: adapting your workplace, moved to another job, adapting your home, adapting your car, customized shoes (yes, no)
Lifestyle habits	Physical exercise	List of 15 physical activities (yes, no and number of hours)
	Visited health spa	Yes, no
	Money spent on rehabilitation	Amount in euros
	Smoking	Non smoker, sporadically/socially, fewer than 10 cigarettes per week, 10–20 cigarettes per week, 21–60 cigarettes per week, over 60 cigarettes per week
	Alcohol	Never, ocassionally, 1–3 times per month, 1–2 times per week, 3–5 times per week, every day
Diagnostic journey	Age of onset of symptoms	Numerical
	Age at diagnosis	Numerical
	HCP who made the diagnosis	List of 4 HCPs
	HCP seen before diagnosis	List of 6 HCPs (yes, no, other)
	First tests for diagnosis	List of 6 medical tests: MRI scan, X-rays, genetic analysis, ultrasound scan, radionuclide scintigraphy, CT scan (yes, no, other)
	Result of HLA-B27	Positive, negative, do not know
	Familiars with axSpA	Kinship and number
Healthcare resource use	Main health insurance	Public, private, out-of-pocket, other
	Number of visits to health professionals in the past 12 months	List of 10 HCPs (numerical)
	Number of medical tests for follow-up in the past 12 months	List of 7 medical tests (numerical)
	Number of inpatient admissions in the past 12 months	Numerical
	Number of uses of emergency services in the past 12 months	List of 4 emergency services: hospital, healthcare centre/outpatient clinic, home emergency, ambulance (numerical)
Treatment	Pharmacological	List of 3 treatments: biological therapy, NSAIDs, and DMARDs (Yes, no)
		Impact on 9 areas (score from 0 to 10)
	Visited health spa	Yes, no
	Money spent on rehabilitation	Amount in euros
	Alternative treatments	Acupuncture, homeopathy, none, other.
	Discussion of treatment goals with HCP	Yes, no
Comorbidities and extra-articular manifestations	Comorbidities associated to axSpA	List of 27 comorbidities: psoriatic arthritis, uveitis, episcleritis, gout, fibromyalgia, spinal or other fractures, liver disease, genital lesions, hypertension, hypercholesterolemia, diabetes, kidney failure, heart failure, cataracts, glaucoma, irregular heart beat, pacemaker fitted, coronary artery disease, atherosclerosis, any severe infection requiring inpatient hospital admission, any severe infections requiring antibiotics, sleep disorders, depression, anxiety, obesity/overweight (Yes, no)
	Extra-articular manifestations	Uveitis and inflammatory bowel disease (ulcerative colitis, Chron’s disease) (Yes, no)
Psychological health	Psychological distress	12-item General Health Questionnaire (GHQ-12) (0–12)
	Presence of anxiey, depression, or sleep disorders	Yes, no
	Visits to psychologists/psychiatrists in the past 12 months	Numerical
Disease outcomes	Disease activity	Bath Ankylosing Spondylitis Disease Activity Index (BASDAI) (0–10)
	Body areas with inflammation	Inflammation in body areas (yes, no)
	Spinal stiffness	Patient-reported stiffness or ankylosis in the spine (yes/no)
		Degree of restriction in cervical, dorsal and lumbar areas (1–4)
		Global Stiffness Index (3–12)
Patient disease-related attitudes and treatment goals	Fears related to axSpA	One open-ended question
	Hopes related to axSpA	One open-ended question
	Treatment goals related to axSpA	One open-ended question

In addition, a range of supplementary indices were collected in the questionnaire to assess specific areas:

1. BASDAI (Bath Ankylosing Spondylitis Disease Activity Index)—a validated self-administered questionnaire assessing disease activity in patients with axSpA; relating to symptoms of fatigue; pain in the spinal column; inflammation/pain in joints other than the neck, back, and hips; areas of localized tenderness (also called enthesitis or inflammation of tendons and ligaments); and the level and duration of stiffness in the morning. Possible scores range from 0 (no activity) to 10 (maximum activity).
2. General Stiffness Index—this index, developed specifically for this study, assesses the degree of stiffness experienced by patients in the spinal column, distinguishing between the cervical, dorsal, and lumbar areas. Possible responses range from the least to the most affected column (1, without stiffness; 2, mild stiffness; 3, moderate stiffness; and 4, severe stiffness), total scores are obtained by adding together the responses in each of the areas of the spine without weighting resulting in a scale ranging from 3 to 12. This index showed an acceptable internal reliability (Cronbach alpha = 0.79).
3. Global Limitation Index—this index, developed specifically for this study, assesses the degree of limitation in 18 activities of daily life (dressing, bathing, showering, tying shoe laces, moving about the house, climbing stairs, getting out of bed, using the bathroom, shopping, preparing meals, eating, household cleaning, walking down the street, using public transportation, driving, going to the doctor, doing physical exercise, having sex). Each of these 18 activities was assigned as 0 for no limitation, 1 low limitation, 2 medium limitation and 3 high limitations, resulting in values between 0 and 54. A total score from 0 and 18 was considered low limitation, between 18 and 36 medium limitation, and between 36 and 54 high limitation. Cronbach alpha of 0.97 demonstrated excellent internal reliability.
4. GHQ-12 (General Health Questionnaire–12)**—**this questionnaire evaluates psychological distress using 12 questions. For the present study, these were transformed into a dichotomous score (0-0-1-1), called the GHQ score, to eliminate any bias resulting from the tendency of the respondents to choose answers 1 and 4 or 2 and 3. The cutoff point of 3 implied those with a score of 3 or more may be experiencing psychological distress [14].

### Sample Selection and Recruitment

The sample selection inclusion criteria were as follows:

- aged ≥ 18 years,
- residents of the specified European country,
- a self-reported diagnosis of axSpA, including Ankylosing Spondylitis or non-radiographic axSpA,
- visit to a healthcare professional for axSpA in the 12 months prior to participation.

Participants were recruited between July 2017 and March 2018 by GfK through their existing database of respondents. In Austria, Norway, Slovenia, Sweden, the Netherlands, Italy, and Russia, Patient Advocacy Groups (PAGs) also supported recruitment by distributing the survey to their members. The questionnaire was completed via an online platform for survey data collection. In addition, the database from the Atlas of Axial Spondyloarthritis in Spain 2017 [15•] was retrospectively added to the EMAS database.

### The EMAS Working Group

The EMAS project is a collaboration led by the Health & Territory Research group of the University of Seville, ASIF, and a steering committee composed of patient representatives and internationally recognized rheumatologists, psychologists, and researchers specialized in axSpA.

## Results

### Participation Rate and Socio-demographics

**Fig. 1:**
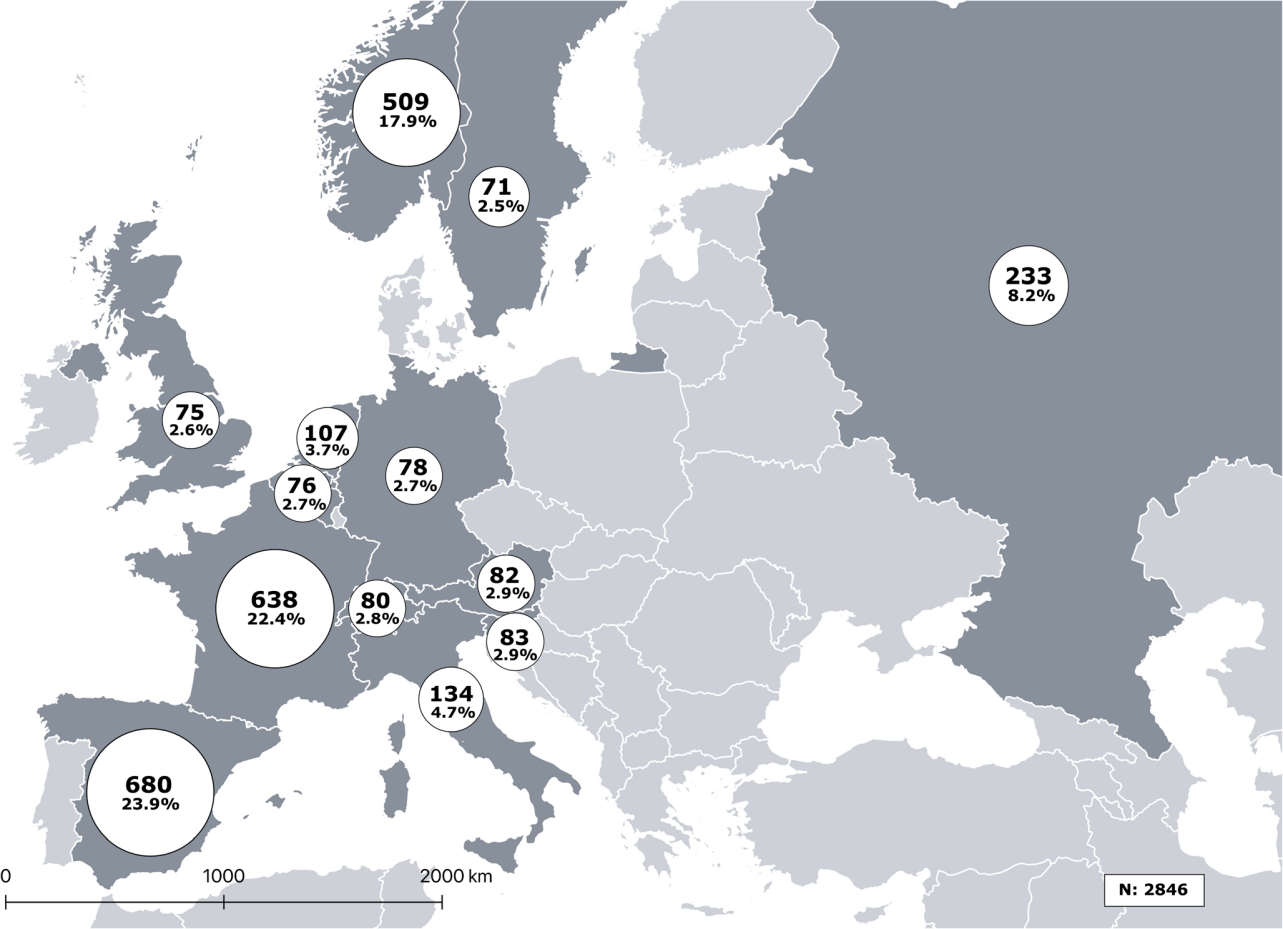
Distribution of EMAS survey participants by European country

A total of 2846 people with self-reported axSpA participated in the EMAS survey. Figure 1 presents the distribution of participants by country, with the largest sample sizes found in Spain, France, Norway, and Russia in that order; EMAS patient socio-demographic, anthropometric characteristics, and lifestyle habits are summarized in Table 2. Overall, three out of five participants were female (61.4%) with a mean (SD) age of 44 (12) years. The majority of participants were either married or in a relationship and were at least high school educated.

**Table 2:** Socio-demographic, anthropometric characteristics, and lifestyle habits

Variable, *n* patients with data available	Mean ± SD/*n* (%)
Age (years) *n* = 2846	43.9 ± 12.3
Gender (female), *n* = 2846	1746 (61.4)
Marital status, *n* = 2846	
Single	601 (21.1)
Married	1933 (67.9)
Separated/divorced	273 (9.6)
Widowed	39 (1.4)
Educational level, *n* = 2846	
No schooling completed	32 (1.1)
Primary school	263 (9.2)
High school	1181 (41.5)
University	1370 (48.1)
Monthly income (euros) per household member, *n* = 2289	1122.6 ± 902.7
BMI, *n* = 2846	
Underweight (< 18.5)	108 (3.8)
Normal weight (18.5–24.9)	1252 (44.0)
Overweight (25–29.9)	953 (33.5)
Obesity (> 30)	533 (18.7)
Smoking, *n* = 2751	
Non smoker	1851 (67.3)
Less than 10 cigarettes/day	380 (13.8)
More than 10 cigarettes	520 (18.9)
Alcohol consumption, *n* = 2751	
Never or occasionally	1810 (65.8)
1–2 times per week	745 (27.1)
More than twice per week	196 (7.1)
Member of a patient support group, *n* = 2846	1107 (38.9)

### Disease-Specific Characteristics

Disease characteristics are depicted in Table 3. The majority of participants reported a diagnosis of AS (79.2%), while the remainder reported being diagnosed with nr-axSpA (8.5%) or just axSpA without specifying the subtype (12.3%). The average age of symptom onset was 26.2 (11.1) years, the mean disease duration was 17.2 (12.2) years, and the mean diagnostic delay reported was 7.4 (8.4) years. Seventy-one percent of those who reported their HLA-B27 status stated that they were HLA-B27 positive. Around 20% of participants reported a diagnosis of an extra-articular manifestation, comprising uveitis or inflammatory bowel disease.

**Table 3:** Disease-specific characteristics

Variable, *n* patients with data available	Mean ± SD/*n* (%)
Type of condition, *n* = 2846	
Ankylosing spondylitis	2254 (79.2)
Non-radiographical axial spondyloarthritis	304 (8.5)
Unspecified axial spondyloarthritis	288 (12.3)
Age at onset of first symptoms, years, *n* = 2721	26.2 ± 11.1
Age at diagnosis, years, *n* = 2722	33.7 ± 11.5
Diagnostic delay, years *n* = 2652	7.4 ± 8.4
Disease duration, years *n* = 2716	17.2 ± 12.4
Extra-articular manifestations, *n* = 2096	
Uveitis	469 (22.4)
Inflammatory bowel disease	294 (14.0)
HLA-B27 (positive), *n* = 1799	1283 (71.3%)
BASDAI (0–10) *n* = 2584	5.5 ± 2.0
Spinal Stiffness Index (3–12), *n* = 2660	7.7 ± 2.5
Maximum degree of stiffness, *n* = 2707	
No stiffness	187 (6.9%)
Mild	471 (17.4%)
Moderate	934 (34.5%)
Severe	1115 (41.2%)
Global Limitation Index (0–54), *n* = 2771	
Overall limitation	20.4 ± 16.3
Low (0–17)	1383 (49.9%)
Medium (18–35)	801 (28.9%)
High (36–54)	587 (21.2%)

The mean BASDAI score was 5.5 (2.0), with the majority of participants reporting at least moderate spinal stiffness and 50.1% reporting medium to high functional limitation during disease flares.

### Working Life, Psychological Health, and Disease-Related Attitudes

Results of the impact of axSpA on working life, psychological health, and patient disease-related perceptions are summarized in Table 4. Nearly half of the participants reported that their disease influenced their job choice and 74.1% reported having difficulties finding a job due to the disease. Additionally, more than half of the participants reported psychological distress (61.5%), with one out of three reporting anxiety and/or depression.

**Table 4:** Working life, psychological health, and disease-related perceptions

Variable, *n* patients with data available	Mean ± SD/*n* (%)
Employment status of labor force, *n* = 1653	
Employed	1457 (87.7)
Unemployed	205 (12.3)
Employment status of economically inactive, *n* = 1042	
Temporary sick leave	304 (29.2)
Permanent sick leave	292 (28.0)
Retired	230 (22.1)
Early retirement	43 (4.1)
Homemaker	114 (10.9)
Student	59 (5.7)
Required a workplace adaptation due to axSpA, *n* = 2651	1163 (43.9%)
AxSpA influenced job choice, *n* = 2527	1156 (45.7%)
Difficulties finding a job due to axSpA, *n* = 2071	1534 (74.1%)
Psychological and sleep comorbidities, *n* = 2096	
Sleep disorder	1058 (50.5)
Anxiety	809 (38.6)
Depression	710 (33.9)
GHQ score, (0–12) *n* = 2640	4.9 ± 4.1
At risk for psychological distress (GHQ ≥ 3), *n* = 2640	1624 (61.5%)
Most common fears, *n* = 2435	
Disease progression	801 (32.9)
Suffering pain	743 (30.5)
Loss of mobility	730 (30.0)
Most common hopes, *n* = 2435	
Stop disease progression	791 (32.5)
Eliminate pain	748 (30.7)
Effective treatments	567 (23.3)
Most common treatment goals, *n* = 2435	
To eliminate/reduce pain	696 (28.6)
To improve mobility	469 (19.3)
To improve my quality of life	202 (8.3)
Talked with your physician about treatment goals, *n* = 2496	1663 (66.6)

Participants commonly reported fear of disease progression, fear of suffering pain, or loss of mobility. Participants’ hopes were mainly to halt disease progression, to eliminate pain, and to receive effective treatment. However, one third of the participants surveyed reported that they had not talked to their clinician about their personal treatment goals.

## Discussion

In this very large European sample, the observed data indicate important unmet needs in axSpA, including long diagnostic delay, deterioration of quality of life, and high burden of disease for patients. First, there is an ongoing and critical need for early and accurate diagnosis. The EMAS diagnostic delay was calculated at over 7 years and confirmed the results of a meta-analysis conducted by Jovaní et al., which found the diagnostic delay to be 8.8 years for females and 6.5 years for males [16]. Furthermore, EMAS results showed that patients on average visited two healthcare professionals, mainly general practitioners (GPs), followed by orthopedic specialists, physiotherapists, and osteopaths (excluding rheumatologists), prior to receiving a diagnosis. It is therefore necessary to improve disease education among healthcare professionals, specifically those responsible for referring patients to a rheumatologist (e.g., primary care physicians, physiotherapists, orthopedic surgeons), as well as optimizing collaboration between them in order to shorten the patient journey to diagnosis, and ultimately effective treatment.

EMAS results also showed a high burden of disease for patients. The majority of participants reported moderate to severe limitation during disease flares, which was especially evident while performing daily activities including physical exercise, cleaning, getting out of bed, or getting dressed. Participants also reported difficulties finding a job due to their axSpA (74.1%), that the disease influenced their job choice (45.7%), and that they required workplace adaptation (43.9%).

As in previous studies [17], the EMAS sample showed a high prevalence of mental health difficulties. 61.5% of the sample was at risk for psychological distress, with 33.8% and 38.6% respectively reporting depression and/or anxiety. This contrasts with the WHO prevalence rates, in which anxiety within European participating countries is reported to be between 3.1% (Russia) and 7.4% (Norway) and depression between 4.5% (UK) and 5.5% (Russia).

Additionally, though previous studies have explored axSpA patient personal hopes and fears related to the disease using quantitative questionnaires [18], EMAS adopted a qualitative approach to understand these factors. When asked to state their disease-related hopes and fears, EMAS participants most frequently reported fear of and hope of stopping disease progression and pain. This is understandable as patients with axSpA suffer from a high degree of anxiety and uncertainty due to the unpredictability of disease flares [5].

These axSpA-related hopes and fears may consequently influence several factors including the patient-physician relationship or treatment adherence [19]; it is critical for patients to share these with their physician. Equally important to the patient-physician dialog is the discussion of the patient’s personal treatment goals. One in three EMAS participants had not discussed their personal treatment goals with their physician. Ultimately, both healthcare professionals and patients should be encouraged to engage in a proactive discussion regarding expectations and goals for axSpA treatment to enable effective shared decision-making and the design of individualized treatment strategies that provide optimal management of the disease [20].

EMAS is the largest survey carried out to date for people with axSpA, across 2846 respondents from 13 European countries. The EMAS focus was on understanding the patient perspective through a holistic approach and utilizing a questionnaire designed for patients, by patients. As such, EMAS collected not only clinical characteristics of the disease but also the impact this had on patient’s psychological health, daily activities, and working and social life as well as how the disease relates to their hopes and fears, all of which are considered relevant and important aspects to patients with axSpA.

We acknowledge that EMAS has some limitations. First, the survey relied on self-reported data, and did not attempt to confirm participant diagnosis nor to support participant responses with clinician reported assessments. As such, clinical data such as the BASDAI or GHQ-12 scores may also suffer from response bias. Nevertheless, the sample characteristics were consistent with previous cohorts including patients with confirmed axSpA [9–12], and as the aim of the survey was to better understand the patient perspective, direct feedback was preferred.

Secondly, we used some non-validated scales or indices for assessing certain factors, such as functional limitations in daily activities and spinal stiffness. The reason for utilizing such scales or composite indices originated during the preliminary phase of the survey development, when patients expressed their concern about not being able to report all aspects of their disease if other scales or indices were to be employed. In any case, a good Cronbach alpha value was obtained for the indices employed in EMAS, which support the reliability of these instruments in this sample. Lastly, the differences in sample sizes between countries, resulting from the two recruitment methods employed (GfK online panel and patient groups), naturally skew the aggregate data towards the experiences of patients in countries with greater sample weight.

Despite these limitations, EMAS adopts a multidisciplinary approach, including the medical and patient community within the research team and aiming to understand the patient experience from a holistic perspective. Results from EMAS were presented at the 13th General Council Meeting of ASIF 2018 held in Guangzhou (China) during which the implications of the findings were discussed with patient and rheumatologist leaders from around the world. They were also disseminated at professional congresses, including EULAR 2018 held in Amsterdam, International Congress on Spondyloarthritis (ICS) 2018 in Ghent, 2018 French Rheumatology Congress (SFR) in Paris, and ACR 2018 in Chicago, in order to enhance interest in better understanding of the patient perspective within the scientific community. Continuing its momentum, the EMAS survey and vision are currently being expanded globally as the International Map of Axial Spondyloarthritis (IMAS), including Canada, the USA, Mexico, Costa Rica, Colombia, Argentina, South Korea, Taiwan, and Turkey. By broadening the scope of the survey outside of Europe, the IMAS project will seek to describe the burden of disease from the perspective of patients around the world.

## Conclusion

By highlighting the important limitations and disease burden that participants face in their daily life, EMAS emphasizes the need to take urgent measures to reduce the burden of disease associated with axSpA by reducing diagnostic delay and ensuring that patients are optimally and holistically managed, including access to therapies such as exercise programs, psychological, and physiotherapeutic care. EMAS also reaffirms the need to incorporate the patient’s perspective into clinical practice, as it facilitates shared decision-making between patients and physicians, which improves disease management, increases patient participation in their care, ensures greater therapeutic adherence, and generates better physical and psychological health outcomes.
